# Hospital Meetings, &c.

**Published:** 1897-02-27

**Authors:** 


### CITY OF LONDON HOSPITAL FOR DISEASES OF THE CHEST.

### HOSPITAL MEETINGS, &c.

### CHARING CROSS HOSPITAL.

The annual court of governors of the Charing Cross Hospital was held in the board-room of the hospital on Wednesday, 17th inst., Lord Glenesk in the chair. The report stated that during the year 1896, 2,119 in-patients, 11,397 out-patients, and 10,170 casualties had been treated. The ordinary income of the hospital during the same period amounted to £10,688, and legacies to £3,618; while the ordinary expenditure amounted to £14,844. The debt at the date of the report was £17,000.

The Chairman, in moving the adoption of the report, congratulated the hospital on its general position. Of course they all knew that it had had to make a special appeal on account of exceptional circumstances, but, so far as the past year was concerned, the hospital had received its fair measure of support. The great need of the hospital was increased accommodation, not as far as its general wards were concerned, but in other ways. They had the site for the extension in that formerly occupied by Toole's Theatre (now pulled down), and if the special appeal was well responded to the funds for carrying out this work would be forthcoming too.

The motion was seconded by Mr. J. S. Pearce and carried. Votes of thanks to the council, the several committees, the medical staff, honorary solicitors, auditors, and the chairman were passed.

The Rev. S. B. C. Beynon, Mr. F. Kynaston Metcalfe, and Lord Kinnaird were re-elected members of the council, the vacancies still remaining on that body being left to the council to fill up.

### CITY OF LONDON LYING-IN HOSPITAL.

The annual court of governors of the City of London Lying-in Hospital was held at the hospital on Wednesday, the 17th inst., Mr. Charles Gordon in the chair. From the report it appears that the past year has been a satisfactory one. The number of women delivered was 493, the highest number treated in the hospital in any of the past thirty years, with the exception of the year 1868, when the same number were attended. Besides this 1,681 women were treated in their own homes, a number only exceeded twice since this department was opened in 1872. The ordinary income amounted to £3,496 and the ordinary expenditure to £3,451. In addition £340 in legacies was received, while £56 was spent on extraordinary repairs and improvements.

The report having been adopted on the motion of the CHAIRMAN, seconded by Mr. G. BERRY, votes of thanks to the committee and staff were passed. Dr. Yarrow was re-elected surgeon-accoucheur for the ensuing year, while Mr. H. Gordon Jeaffreson was elected a member of the committee in place of Mr. Arthur Hay.

### ROYAL FREE HOSPITAL.

The annual Court of Governors of this hospital was held on Thursday, the 18th inst., Mr. Justice BRUCE, chairman of the committee, presiding. The report stated that, as a consequence of the reopening of the wards which had been closed during the rebuilding operations and of the improved accommodation afforded by the completion of the hospital buildings, there had been a considerable increase in the work done. During the past year 2,070 in-patients and 38,992 out-patients had been treated, a number largely in excess of any yet recorded. The Council of the School of Medicine for Women had decided to incorporate the school under the title of the Royal Free Hospital School of Medicine for Women. This the committee has agreed to on certain conditions.

The CHAIRMAN, in moving the adoption of the report, said that the out-patient departments of hospitals were attracting a great deal of attention at the present time. At the Royal Free Hospital, however, while a large number of out-patients had been treated, it was satisfactory to find that there had been no abuse whatever. At that hospital they had the advantage of having the assistance of one of the almoners of the Society for the Relief of Distress, who investigated the cases which attended the out-patient department in order to ascertain whether the persons seeking relief were persons who should properly be relieved, and were unable to afford to pay for medical attendance. As a result of the inquiries made, it was found that out of 2,475 cases investigated only seven were cases where the patients could possibly have paid for medical relief themselves. The committee found it increasingly difficult year after year to keep pace with the ever-growing expenditure, and they had intended during the present year to make a special appeal for funds. Taking into consideration, however, the number of other appeals which had been started, it had been decided to put this off until next year.

Mr. CHARLES BURT, chairman of the Weekly Committee, seconded, and drew attention to the fact that the whole of the cash balance in hand at the close of 1896 would be exhausted by the end of March. Unless the hospital got substantial help before that time they would have to get into debt, a state of affairs almost unknown to that institution. He also pointed out that the hospital required subscriptions to the amount of £5,000 a year in order to carry on its work in the future as efficiently as it had done in the past.

The report was adopted, and votes of thanks to the various committees and officers concluded the proceedings.

### LONDON FEVER HOSPITAL.

The annual meeting of the governors of this hospital was held on Friday, the 19th inst., at the Hotel Victoria, Northumberland Avenue, Lord BALFOUR of BURLEIGH presiding.

Mr. J. HAMER, chairman of the Finance Committee, presented the financial statement for the year, saying that the most hopeful sign of the balance sheet was the fact that the ordinary receipts were substantially above the ordinary expenditure.

The report of the Council was adopted on the motion of Dr. CAYLEY.

The CHAIRMAN, in moving a resolution approving of the proceedings of the committee during the year, said that, from the reports which had been placed before the meeting, it would be seen that 874 patients had been treated, of whom 135 were servants of governors or employés of firms, clubs, or hotels who paid subscriptions as governors according to scale, and, therefore, were entitled to the free treatment of their servants. The remaining 739 paid fees amounting in the whole to one-fourth the cost incurred in treating them. It was also satisfactory to note that the death-rate for scarlet fever in the hospital had only been 1·3 per cent. as compared with an average death-rate of 3·6 per cent. among the cases of scarlet fever notified throughout London generally. During the past two years £20,000 had been spent on rebuilding the hospital—£10,000 the year before last and £10,000 last year—and the committee would like to spend at least as much again to rebuild the wards which were not up to date. Whether, however, they could do that in the near future or not, and whether they could build their convalescent home which for some years had been under consideration must depend upon the response which was given to their appeal. They had had to their great regret to reject 184 patients who were sent to the hospital, but they believed that if the rebuilding of the hospital could be completed and the convalescent home finished they would be able to meet all reasonable demands which were likely to be made upon the institution. He drew attention to the fact that up to the present time the receipts for 1897 were £700 worse than they were at the same date in 1896, the cause of this being, in his opinion, the number of special funds which had been started this year. While believing that the Prince of Wales's Hospital Fund—which had been most judiciously thought out—would, if taken up properly, do a great amount of good, and would stimulate the interest taken in the hospitals of London, he yet wished to point out to the old and established friends of the hospital that if they simply transferred to that fund the money which they had hitherto given to the hospital, they were not only doing no good but were doing a lot of harm to the institution itself. The Prince of Wales's Fund, to do good to the hospitals of London generally, must be something in excess of what had been given in the past, and not a mere substitution, or sending to a central fund what had hitherto been sent to the individual funds of the metropolis. He hoped that what he had said was not calulated to give offence to anybody, as it was not meant for more than a friendly warning which he had reason to believe was needed in the interest of every institution in London.

Lord BALFOUR was re-elected president of the hospital, and the meeting concluded with the usual votes of thanks.

### QUEEN CHARLOTTE'S HOSPITAL.

The annual meeting of Queen Charlotte's Hospital was held in the board-room of the hospital on Monday, the 22nd inst., Major MACMAHON in the chair. The report stated that during the year 1896 1,151 patients had been delivered in the hospital, an increase of 27 on the previous year, and the largest number ever received in one year. There were ten deaths amongst the in-patients, the rate of mortality being 8·68 per thousand. In the out-patient department 1,122 patients were delivered, being a decrease compared with 1895, to a considerable extent due to the removal of many poor people from the neighbourhood of the hospital, in consequence of the pulling down of a large number of small houses for the Manchester, Sheffield, and Lincolnshire Railway Extension to London. The ordinary income amounted to £3,610, and legacies to £443; while the ordinary expenditure was £4,273, and the extraordinary expenditure £60. During the year the committee had been able to formulate a scheme for rebuilding the hospital. To carry out the entire scheme the sum of £12,000 was needed, and in response to the special appeal issued, upwards of £4,000 had already been received. The report was adopted, and the election of the committee and auditors, and the passing of votes of thanks to the medical staff and the chairman completed the business of the meeting.

### CITY OF LONDON HOSPITAL FOR DISEASES OF THE CHEST.

The annual court of governors of this institution was held on Thursday, the 18th inst., Mr. Julian Hill, treasurer, presiding. The Chairman, in moving the adoption of the report, called attention to the increase of the work of the hospital during the past year. The number of in-patients was 939 and of out-patients 16,166. The death of Sir J. Eric Erichsen deprived the hospital of his valuable services as consulting surgeon. Lord Lister had, however, consented to accept the vacant office, and the committee felt that no greater appreciation of the importance of the hospital could be shown than by its having secured the countenance and aid of one of the most distinguished men of the day. The expenditure, £11,933, has exceeded the income by £2,430. To meet this deficit a loan secured on the funded property of the hospital has had to be resorted to.

### CLERGY ORPHAN CORPORATION.

The annual court of this society was held on Tuesday, the 23rd inst., at the Church House, Westminster, the Earl of Cranbrook, G.C.S.I., in the chair. Amongst those present were Archdeacon Smith, Sir Paget Bowman, Bart., Lord Thring, the Rev. Prebendary Whittington, Canon Bailey, Colonel Victor Milward, M.P., Mr. T. H. Thornton, C.S.I., and the Rev. Canon Elwyn. The report stated that the income from voluntary sources had increased from £7,508 in 1895 to £7,773 in 1896. The committee had proceeded with the extension of the school building at Canterbury, and the erection of a new wing for the accommodation of thirty boys and for the purposes of a junior department, was begun last summer, and would be finished, it was hoped, in the course of this year. The girls' school at Merry Hill, Bushey, which had been in process of construction during the last two years, is now ready for occupation, and the removal from Windsor is in progress. The report concluded with the statement that the fact that at least two-thirds of the children in the schools and of the numerous candidates waiting for admission were the orphans of beneficed clergymen proved that, through the continuous decrease in the value of livings during the last few years, the clergy were less able year by year to provide adequately for their families.

The Chairman, in moving the adoption of the report, referred in feeling terms to the death of the late Archbishop of Canterbury, who had occupied the position of president of the society. All who had come in contact with him had felt what an atmosphere of brightness and hope there always was around him.

Colonel Victor Milward, M.P., seconded, and the report was adopted.

It was decided that not less than twelve boys and ten girls be elected into the schools in May next.

The election of officers and the passing of votes of thanks concluded the business.

